# Higher Habitual Nuts Consumption Is Associated with Better Cognitive Function among Qatari Adults

**DOI:** 10.3390/nu13103580

**Published:** 2021-10-13

**Authors:** Hajer Nafea, Omnia Abdelmegid, Sara Qaddourah, Zainab Abdulwahab, Joyce Moawad, Zumin Shi

**Affiliations:** Human Nutrition Department, College of Health Sciences, QU Health, Qatar University, Doha 2713, Qatar; hn1604058@student.qu.edu.qa (H.N.); oa1601767@student.qu.edu.qa (O.A.); sq1703090@student.qu.edu.qa (S.Q.); za1601366@student.qu.edu.qa (Z.A.); jmoawad@qu.edu.qa (J.M.)

**Keywords:** nuts consumption, cognition, mean reaction time, adults, Qatar Biobank

## Abstract

The association between nuts intake and cognitive function is inconclusive. We aimed to investigate the association between habitual nuts consumption and cognition among Qatari adults. Data from 1000 participants aged >20 years who attended Qatar Biobank (QBB) were used. Nuts consumption was assessed by a food frequency questionnaire (FFQ). Blood samples were measured for magnesium, lipids and glucose. Mean reaction time (MRT) was used as an indicator of cognitive function. Linear regression was used to assess the association. A total of 21.1% of the participants reported consuming nuts ≥4–6 times/week (high consumption) while 40.2% reported consuming ≤1 time/month (low consumption). The mean MRT was 715.6 milliseconds (SD 204.1). An inverse association was found between nuts consumption and MRT. Compared to those with a low consumption, high consumption of nuts had a regression coefficient of −36.9 (95% CI −68.1 to −5.8) after adjusting for sociodemographic and lifestyle factors. The inverse association between nuts and MRT was mainly seen among those >50 years. There was an interaction between nuts consumption and hypertension. The association between nuts consumption and MRT was not mediated by hypertension, diabetes, or serum magnesium. Habitual higher consumption of nuts is positively associated with cognitive function, especially among old adults.

## 1. Introduction

Globally, cognitive impairments including Alzheimer’s disease and dementia have become a major concern. It has been shown that 42% of the worldwide population aged ≥60 years were affected by mild cognitive impairment (MCI) [1]. The total number of people with dementia has remarkably increased from 20.2 million in 1990 to 43.8 million in 2016 with women being more affected than men [2]. Dementia was the fifth leading cause of death and accounted for 2.4 million deaths worldwide in 2016 [3]. It has been estimated that a third of dementia cases can be prevented or delayed by interventions to reduce associated risk factors including unhealthy diet, hypertension, diabetes, obesity and depression [4].

The role of diet on the prevention and management of cognitive impairment has been studied in animal studies as well as in human studies [5]. Diet can affect brain function through different mechanisms including the regulation of neurotransmitter pathways, synaptic transmission, membrane fluidity and signal transduction pathways. Many nutrients have been examined for their role in cognitive function (e.g., omega-3 fatty acids, flavonoids, vitamin E) [5]. Nuts are good sources of polyunsaturated fatty acids, phytosterols, polyphenols, and magnesium. Nuts consumption has been shown to be beneficial for the prevention of many chronic diseases including diabetes and hypertension [6]. Since hypertension and diabetes are risk factors for cognitive impairment, it is possible that nuts consumption can improve cognitive function. Few population observational studies have examined the association between nuts consumption and cognitive function. Findings from most of these studies suggest a beneficial effect of nuts on cognition [7,8,9,10]. For instance, a study conducted on 4822 adults in China found that higher nuts consumption may contribute to a better overall cognition at older ages [9]. On the other hand, few studies showed there is no association between cognitive function and nuts intake [11,12]. For instance, a randomized cross-over trial showed no effect of walnut supplementation on memory, non-verbal reasoning, and mood in young adults [11].

Findings from animal studies suggest a beneficial effect of magnesium on cognitive function [13,14,15]. In human studies, serum magnesium and magnesium intake have been shown to be positively related to cognitive function [16]. It is unknown whether serum magnesium mediates the association between nuts consumption and cognitive function.

Based on the National Household Income Expenditure Survey 2012–2013 conducted among households in Qatar, on average each Qatari purchased 28 g of legumes and nuts per day [17]. As nuts consumption is part of the food culture in Qatar, it is feasible to assess the association between nuts consumption and cognitive function. 

The aims of the current study were to (1) to assess the association between nuts consumption and cognitive function among the Qatari population; (2) to test whether magnesium, hypertension, and diabetes mediate this association between nut consumption and cognitive function.

## 2. Materials and Methods

### 2.1. Study Design and Sample

Qatar Biobank Study (QBB) is an ongoing cohort study [18]. The baseline of the study started in 2012. By 2018, a total of 15,000 participants were enrolled. QBB gives Qatar University students’ project free access to the data of 1000 participants. In the current analysis, 1000 Qatari adults aged 20 years and above were randomly selected by the QBB management team. Using a self-administered questionnaire, socio-demographic information, lifestyle factors, and dietary habits were collected. In addition, medical and family history information were obtained by a nurse interview and a health examination was conducted at Hamad Medical Center, Doha. All protocols of the QBB study were approved by the Hamad Medical Corporation Ethics Committee in 2011 and QBB Institutional Review Board (IRBs) from 2017 onward. The current study was approved under exemption category (Ex-2019-RES-ACC-0163-0086) by the Qatar Biobank.

### 2.2. Outcome Variable: Cognitive Function as Indicated by Mean Reaction Time

Cognitive function was assessed by a computer-based test to measure the mean reaction time (MRT) [19,20]. A visual stimulus was used for the evaluation of MRT. In addition to MRT, a paired episodic memory test was conducted. However, the memory test was not used in the current analysis because the variation is very small.

### 2.3. Exposure Variable: Nuts Consumption

A 102 items food frequency questionnaire (FFQ) was used to assess the dietary habits. The FFQ asked participants to report their usual intake of nuts (never or rarely, once a month, 2–3 times per week, once a week, 4–6 times per week, once per day, ≥2 times/day). In the analysis, nuts consumption was recoded into four categories: ≤1 time/month, 1–3 times/month, 1–3 times/week, and ≥ 4–6 times/week.

### 2.4. Covariates

Age, sex, education, and literacy levels, smoking (non-smokers, ex-smokers, and current smokers), leisure time physical activity level [metabolic equivalent of task (MET), recoded as tertiles], and BMI (overweight is BMI of 25.0–29.9 kg/m^2^ and obesity is BMI of ≥30 kg/m^2^) were used as covariates. Medication use for diabetes and hypertension was collected in interviews by nurses and recoded as “yes” or “no”.

Body height and weight were measured and recorded. Diabetes was defined as HbA1c ≥6.5%, random blood glucose ≥11.1 mmol/L, fasting blood glucose ≥7 mmol/L, or self-reported doctor-diagnosed diabetes [21]. Hypertension was diagnosed as systolic blood pressure >140 mmHg and/or diastolic blood pressure >90 mmHg or previous doctor diagnosis. Serum magnesium was measured at Qatar biobank central lab using the automated colorimetric method. The variations coefficients are 0.3–0.8%. Serum magnesium <0.85 mmol/L is used to characterize subclinical magnesium deficiency.

### 2.5. Statistical Analysis

A Chi-square test was used to compare the differences between different levels of nuts consumption for categorical variables while ANOVA was used for continuous variables. Three multivariable linear regression models were used to assess the association between nuts intake and MRT. Model 1 was adjusted for gender and age; model 2 was further adjusted for smoking, education, leisure time physical activity levels, and intake of fruit and vegetable; model 3 further adjusted for BMI, hypertension, diabetes, and the use of hypertension and diabetes medications. As serum magnesium was not related to nuts consumption, it was not adjusted in the model. In sensitivity analyses, we adjusted for herbal tea and regular coffee consumption, as they were associated with MRT in our previous study [22]. The multiplicative interaction between nuts consumption and chronic diseases (diabetes and hypertension) in relation to MRT was tested by adding the product term of the two in the linear regression model. In Stata, a command (marginsplot) was used to visualize the interaction. Structure equation model was used to test the direct and indirect effect. As nuts consumption was not associated with blood lipids in this study (data not shown), we did not adjust for blood lipids (i.e., LDL, HDL, and TG) as covariates in the multivariable model. Furthermore, there was no interaction between nuts consumption and blood lipids. All analyzes were carried out using STATA (version 16, Stata Corporation, College Station, TX, USA). We considered *p* values < 0.05 (two-tailed) as statistically significant.

## 3. Results

### 3.1. Sample Description

The mean age of the sample was 35.8 (SD 10.3) years (Table 1). In the sample, 21.2% of the participants consumed nuts ≥4–6 times/week while 40.2% consumed nuts ≤1 time/month. Nuts consumption was positively associated with age, fruit and vegetable consumption, and dietary supplement use. Across the levels of nuts consumption, there were no differences in smoking, physical activity, BMI, and serum magnesium. The overall levels of leisure time physical activity in this population were low. Half of the study participants were males. The sample mean of the mean reaction times (MRT) was 715.4 milliseconds (204.1 SD). Nuts consumption was positively associated with regular tea, herbal tea, and Arabic coffee consumption. 

Age was positively associated but education was inversely associated with MRT (Figure S1). Women had a higher MRT than men.

### 3.2. Association between Nuts Consumption with MRT

Nuts consumption was inversely associated with MRT. After adjusting for age, gender, education, smoking, PA, intake of fruits and vegetables, across nuts consumption of ≤1 time/month, 1–3 times/month, 1–3 times/week, and ≥4–6 times/week, the regression coefficients (95%CI) for MRT were 0 (reference), −24.5 (−56.9 to 7.9), −24.0 (−54.0 to–5.9), and −36.9 (−68.1 to −5.8) (p for trend 0.016) (Table 2). After further adjusting for BMI, hypertension, diabetes, and medication use, the above association became weaker but remained statistically significant. In sensitivity analyses, the association between nuts consumption and MRT was independent of herbal tea or regular coffee consumption. 

**Figure 1:**
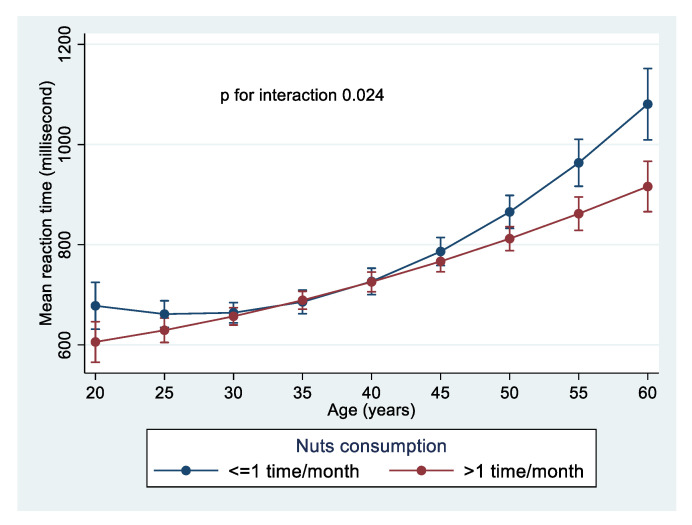
Interaction between nuts intake and age in relation to mean reaction time. Model was adjusted for age, sex, education, and literacy levels, smoking, physical activity level, and BMI.

Figure 1 showed a significant interaction (*p* = 0.024) between nuts intake and age in relation to MRT. Among the older age group, those who had nuts consumption >1 time/month had a lower MRT than those consumed nuts ≤1 time/month. The beneficial effect of nuts consumption was clearly observed among individuals aged above 50 years old. However, among younger participants, there was no such association.

**Figure 2:**
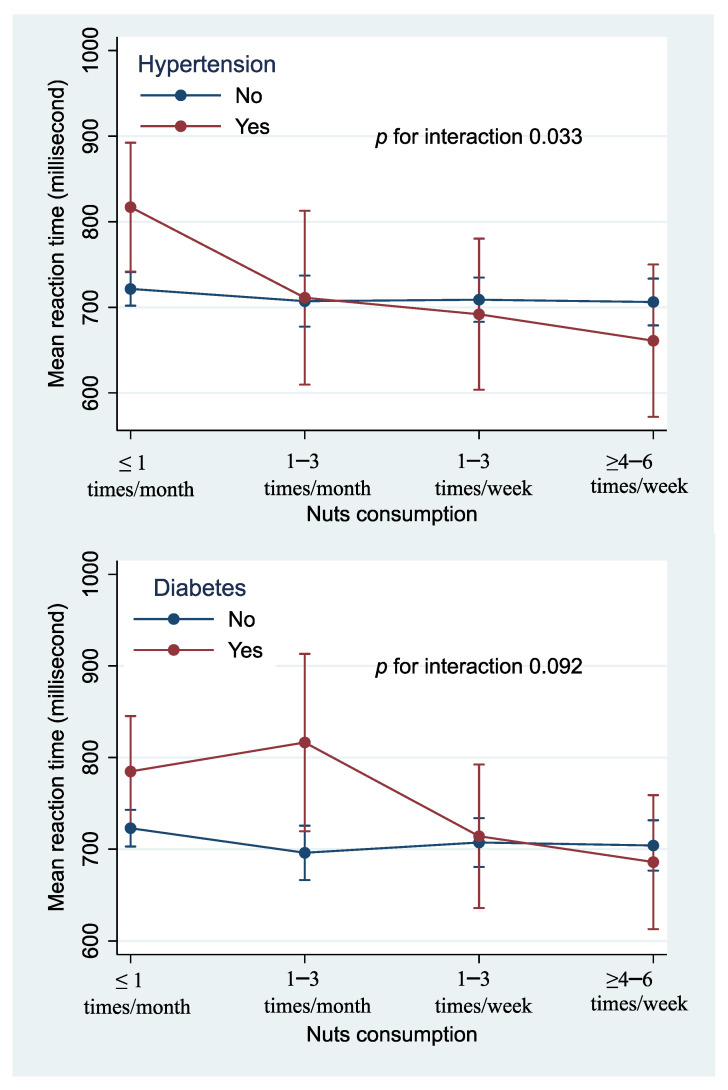
Interaction between nuts consumption and hypertension and diabetes in relation to cognitive function. Models were adjusted for age, sex, education, and literacy levels, smoking, physical activity level, and BMI.

There was a borderline significant interaction (*p* = 0.092) between nuts consumption and diabetes in relation to MRT (Figure 2). Participants with a low consumption of nuts tended to have a longer MRT. However, no association between nuts and MRT was found among those without diabetes. There was a significant interaction between nuts consumption and hypertension. The beneficial effect of nuts on MRT was only seen among those with hypertension.

## 4. Discussion

In this cross-sectional study, nuts consumption was inversely associated with MRT. There was an interaction between nuts consumption and age, hypertension, and diabetes. The inverse association between nuts consumption and MRT was mainly seen among those with an old age, with hypertension, or diabetes. The association between nuts consumption and MRT was not mediated by serum magnesium, hypertension, or diabetes. 

The findings of our study were consistent with the results of most of the previous studies in both humans and animals [7,8,9,10,11,12,13,14,15,16,23,24,25,26]. A cross-sectional study conducted in Spain on 447 participants found that increased intake of Mediterranean-diet components including walnuts is linked to better cognitive function and increased working memory in elderly at high cardiovascular risk [27]. A prospective cohort study of 2613 elderly participants found that high nuts consumption was associated with a better memory, speed, flexibility, and global cognitive score [28]. A prospective cohort study conducted in China (n = 4822) found that consuming greater than 10 g/d of nuts is linked with a higher cognition score and a lower risk of having declined cognitive function [9]. On the other hand, some studies did not find any association between nuts consumption and cognitive function. For example, in a cross-sectional study conducted in Norway, Nurk et al. found that among 2031 males and females aged between 70–74 years there was no significant relationship between nuts consumption and executive function and semantic memory [7]. In the Women’s Health Study, among 6174 women aged 75+ years, nuts consumption was not associated with global cognition and verbal memory during a 5-year follow-up [12]. The inconsistent findings may be due to the study design and different levels of nuts consumption as well as the age of participants. In our study, few participants were aged above 65 years.

The interaction between age, hypertension/diabetes, and low nuts intake is intriguing. Hypertension and diabetes are known risk factors for cognitive impairment. If nuts are beneficial for cognitive function, these effects would be more likely to be observed among those who are at risk for cognitive impairment. It may be beneficial to have an adequate intake of nuts especially in older people, and those with hypertension. 

### 4.1. Clustering of Tea, Coffee and Nuts Consumption

Nuts consumption is part of the food culture and lifestyle. The types of nuts consumption vary by country. In China, peanuts are the most commonly consumed nuts, followed by chestnut, sunflower seed, pumpkin seed, and walnut [9]. In Arabic countries, hazelnuts, pistachio, and pine tree nuts are commonly consumed. In our study, nuts consumption was positively associated with herbal tea and Arabic coffee consumption. In Qatari culture, it is popular that people eat different types of nuts with Arabic coffee or herbal tea as a snack. Based on our findings, tea and coffee consumption is common among Qatari adults. In a previous study conducted in Qatar, there was a positive association between regular coffee and tea consumption with cognitive function [22]. Furthermore, there was an interaction between tea/coffee consumption patterns and LDL in relation to MRT. A Western coffee consumption pattern was inversely associated with MRT among those with a low LDL. It is possible that the association between nuts consumption and MRT was also partly due to a clustering of eating habits. However, in the current study additional adjustment for tea and coffee consumption did not change the association between nuts consumption and MRT. Unlike the interaction between tea/coffee drinking patterns and LDL, there was no interaction between nuts consumption and lipids in relation to MRT. Thus, the mechanisms linking nuts consumption and MRT may not involve blood lipids. 

### 4.2. Potential Physiological Mechanisms

As proposed by previous studies, several mechanisms may explain the association between nuts consumption and cognitive function [8,10,16]. First, sufficient nuts consumption is beneficial for the prevention and management of chronic conditions including diabetes and hypertension, which are both considered risk factors of cognitive decline [29,30]. Second, nuts contain high levels of antioxidants and can reduce the Aβ-induced oxidative stress [31], cell damage, and levels of inflammation [32]. These antioxidants can neutralize the reactive oxygen species (ROS) and decrease the risk of cognition impairment [33]. Moreover, nuts consumption can reduce the risk of cardiometabolic dysfunction and maintain endothelial function [7].

### 4.3. Limitations and Strengths

The study has some limitations. First, MRT is the only indicator used for measuring cognitive function. Other aspects of cognitive functions were not measured. Second, the sample is relatively small and most of the participants were young; therefore, results cannot be extrapolated over the general population. Third, the food frequency questionnaire used included only the frequency of the nuts consumption without specifying the amount or type of the consumed nuts. Thus, we could not estimate the actual amount (grams/day) of nuts intake. In addition, recall bias is considered another limitation in this study. However, this study has several strengths. In the study, a variety of potential confounding factors were measured and adjusted. Chronic conditions were measured including diabetes and hypertension. There is an adequate variation of nuts consumption.

### 4.4. Implications of the Study

This study provides additional information on the association between nuts consumption and cognitive function. Qatar has high rates of chronic non-communicable diseases such as diabetes and hypertension [34], which increase the risk of cognitive impairment [35]. Hypertensive patients with low nuts intake tend to have a poor cognition function in our study. As nuts are an essential part of the Qatari diet [17], promoting nuts consumption in order to prevent chronic disease, including cognitive impairment, is feasible.

## 5. Conclusions

Nuts consumption is beneficial for cognitive function as measured by MRT, especially among those with old age, diabetes, and hypertension. Further prospective studies and randomized clinical trials are needed to assess the amount and type of nuts consumption on cognitive function.

## Figures and Tables

**Table 1:** Baseline sample characteristics by levels of nuts consumption: Qatar Biobank Study (n = 1000).

Factor	≤1 Time/Month (n = 402)	1–3 Times/Month (n = 168)	1–3 Times/Week (n = 218)	≥4–6 Times/Week (n = 212)	*p*-Value ^1^
Magnesium (mmol/L)	0.83 (0.06)	0.84 (0.06)	0.83 (0.06)	0.83 (0.06)	0.22
Age	34.4 (9.9)	36.0 (10.7)	36.3 (9.7)	38.0 (10.9)	<0.001
Gender					<0.001
Male	189 (47.0%)	80 (47.6%)	137 (62.8%)	94 (44.3%)	
Female	213 (53.0%)	88 (52.4%)	81 (37.2%)	118 (55.7%)	
Education					0.028
Low	140 (34.9%)	64 (38.1%)	80 (36.9%)	54 (25.5%)	
High	261 (65.1%)	104 (61.9%)	137 (63.1%)	158 (74.5%)	
Smoking					0.20
Non	272 (67.7%)	123 (73.2%)	133 (61.0%)	145 (68.4%)	
Smoker	79 (19.7%)	27 (16.1%)	46 (21.1%)	35 (16.5%)	
Ex-smoker	51 (12.7%)	18 (10.7%)	39 (17.9%)	32 (15.1%)	
Leisure time physical activity (MET hours/week)	4.5 (16.2)	7.7 (21.2)	6.7 (19.1)	7.9 (34.1)	0.23
BMI (kg/m^2^)	28.2 (6.1)	27.9 (5.3)	28.8 (5.7)	28.0 (5.3)	0.40
BMI categories					0.53
Normal	127 (31.6%)	49 (29.2%)	55 (25.2%)	62 (29.2%)	
Overweight	141 (35.1%)	68 (40.5%)	86 (39.4%)	87 (41.0%)	
Obese	134 (33.3%)	51 (30.4%)	77 (35.3%)	63 (29.7%)	
Supplement use	239 (59.5%)	96 (57.1%)	129 (59.2%)	150 (70.8%)	0.017
Vitamin D and Calcium use	150 (37.3%)	60 (35.7%)	80 (36.7%)	93 (43.9%)	0.30
Vegetable intake (times/week)	14.8 (13.0)	16.0 (13.3)	17.9 (13.0)	22.3 (15.7)	<0.001
Fruit intake (times/week)	5.6 (5.7)	5.4 (5.8)	7.2 (5.6)	9.8 (6.9)	<0.001
Tea and coffee drinking (times/week)					
Tea	6.1 (7.4)	6.4 (7.6)	6.2 (7.4)	8.0 (8.0)	0.023
Herbal tea	2.5 (4.6)	3.0 (4.8)	3.3 (5.2)	4.3 (6.0)	<0.001
Karak	5.8 (6.8)	5.2 (6.2)	6.1 (6.8)	5.0 (6.6)	0.26
Arabic coffee	7.3 (7.8)	7.0 (7.4)	8.2 (7.8)	9.9 (8.2)	<0.001
Instant coffee	3.1 (5.3)	2.8 (5.2)	3.1 (4.9)	3.2 (5.0)	0.84
Regular coffee (e.g., cappuccino)	2.4 (4.9)	1.5 (3.7)	2.1 (4.2)	2.6 (5.1)	0.11
HbA1C (%)	5.5 (0.9)	5.5 (0.8)	5.6 (1.1)	5.6 (0.9)	0.69
Hypertension	37 (9.2%)	15 (8.9%)	21 (9.6%)	23 (10.8%)	0.91
Diabetes	44 (11.5%)	15 (9.3%)	29 (13.7%)	28 (13.6%)	0.52
Insulin use	6 (1.5%)	3 (1.8%)	7 (3.2%)	3 (1.4%)	0.45
Diabetes medication other than insulin	19 (4.7%)	6 (3.6%)	17 (7.8%)	13 (6.1%)	0.26
Supplement use	239 (59.5%)	96 (57.1%)	129 (59.2%)	150 (70.8%)	0.017
Hypertension medication use	23 (5.7%)	8 (4.8%)	9 (4.1%)	15 (7.1%)	0.57
Mean reaction time (millisecond)	721.2 (221.9)	711.2 (214.9)	703.5 (169.7)	719.5 (193.2)	0.75

^1^*p* from ANOVA for continuous measures or chi-square tests for categorical ones.

**Table 2:** Association (β 95% CI) between nuts consumption and cognitive function (mean reaction time) among adults attending Qatar Biobank Study (n = 1000) ^1^.

	≤1 Time/Month	1–3 Times/Month	1–3 Times/Week	≥4–6 Times/Week	*p* for Trend
Model 1 ^2^	0.00	−22.6 (−55.4 to 10.3)	−18.8 (−49.1 to 11.5)	−34.2 (−64.8 to −3.7)	0.030
Model 2 ^3^	0.00	−24.5 (−56.9 to 7.9)	−24.0 (−54.0 to 5.9)	−36.9 (−68.1 to −5.8)	0.016
Model 3 ^4^	0.00	−20.4 (−53.2 to 12.5)	−18.9 (−49.3 to 11.6)	−31.3 (−62.9 to 0.2)	0.048
Sensitivity analyses ^5^					
Model 2 + herbal tea	0.00	−22.6 (−54.8 to 9.7)	−22.1 (−52.0 to 7.7)	−34.2 (−65.3 to −3.2)	0.026
Model 2 + regular coffee	0.00	−25.7 (−58.0 to 6.5)	−24.3 (-54.1 to 5.6)	−36.3 (−67.2 to −5.3)	0.017

^1^ Values are regression coefficients (95% CI) from multivariable linear regression. ^2^ Model 1 adjusted for age and gender. ^3^ Model 2 further adjusted for education, smoking, physical activity, intake of fruit and vegetable. ^4^ Model 3 further adjusted for BMI, diabetes, hypertension, and medication for diabetes and hypertension. ^5^ In sensitivity analyses, herbal tea or regular coffee consumption was adjusted as continuous variables (times/week).

## Data Availability

The current research uses data from the Qatar Biobank Study. Data described in the manuscript, and code book are available by submitting a request to Qatar Biobank study management team at https://www.qatarbiobank.org.qa/ (accessed on 10 September 2021).

## Supplementary Materials

The following are available online at https://www.mdpi.com/article/10.3390/nu13103580/s1, Figure S1: Association between sociodemographic and lifestyle factors with cognitive function.
