# Supplementary material for: Higher Habitual Nuts Consumption Is Associated with Better Cognitive Function among Qatari Adults
Source: Nutrients. 2021 Oct 13;13(10):3580. doi: 10.3390/nu13103580 (PMC8538871; doi:10.3390/nu13103580)
Supplement: Supplementary file 1 [file nutrients-13-03580-s001.zip › nutrients-1403138-supplementary.pdf]

Figure S1 Association between sociodemographic and lifestyle factors with cognitive function

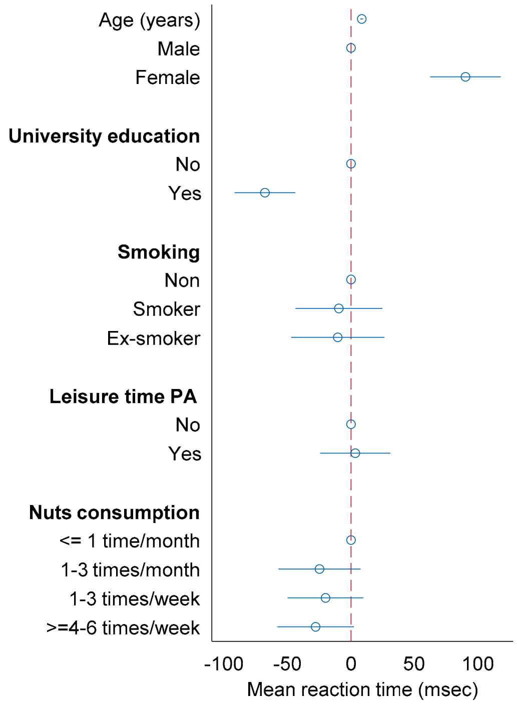

All the variables in the figure were adjusted in the multivariable regression model.
